# SOX2 expression correlates with lymph-node metastases and distant spread in right-sided colon cancer

**DOI:** 10.1186/1471-2407-11-518

**Published:** 2011-12-14

**Authors:** Jens Neumann, Fiorina Bahr, David Horst, Lydia Kriegl, Jutta Engel, Raquel Mejias Luque, Markus Gerhard, Thomas Kirchner, Andreas Jung

**Affiliations:** 1 Pathologisches Institut, Ludwig-Maximilians-Universität München, Munich, Germany; 2 Dana-Farber Cancer Institute, Harvard Medical School, Boston, USA; 3 Tumorregister München des Tumorzentrums München am Institut für medizinische Informationsverarbeitung, Biometrie und Epidemiologie der Ludwig-Maximilians-Universität München, Munich, Germany; 4 Institut für Medizinische Mikrobiologie und Immunologie, Technische Universität München, Munich, Germany

**Keywords:** SOX2, β-catenin, Colon cancer, Metastasis

## Abstract

**Background:**

The transcription factor SOX2, which is involved in the induction of pluripotent stem cells and contributes to colorectal carcinogenesis, is associated with a poor prognosis in colon cancer (CC). Furthermore, SOX2 is a repressor of the transcriptional activity of β-catenin in vitro. Since the majority of CC develop via an activation of the Wnt/β-catenin signalling pathway, indicated by nuclear expression of β-catenin, we wanted to investigate the expression patterns of SOX2 and β-catenin and correlate them with the occurrence of lymph node and distant metastases as indicators of malignant progression.

**Methods:**

The expression of SOX2 and β-catenin was investigated in a case control study utilizing a matched pair collection (N = 114) of right-sided CCs with either corresponding distant metastases (N = 57) or without distant spread (N = 57) by applying immunohistochemistry.

**Results:**

Elevated protein expression of SOX2 significantly correlated with the presence of lymph node- (*p *= 0.006) and distant metastases (*p *= 0.022). Nuclear β-catenin expression correlated significantly only with distant metastases (*p *= 0.001). Less than 10% of cases showed a coexpression of high levels of β-catenin and SOX2. The positivity for both markers was also associated with a very high risk for lymph-node metastases (*p *= 0.007) and distant spread (*p *= 0.028).

**Conclusion:**

We demonstrated that increased expression of either SOX2 or nuclear β-catenin are associated with distant metastases in right-sided CC. Additionally, SOX2 is also associated with lymph-node metastases. These data underline the importance of stemness-associated markers for the identification of CC with high risk for distant spread.

## Background

Colon cancer (CC) is one of the most common human malignancies and one of the major causes of cancer related death worldwide [1]. About 60-80% of CCs develop on the basis of a dysregulation of the Wnt/β-catenin signalling pathway triggered in most cases by mutations in the tumour suppressor gene APC (adenomatous polyposis coli) which is indicated by an accumulation of β-catenin in the tumour cells [2-6]. By immunohistochemistry it was shown that accumulation of nuclear β-catenin correlated with the expression of β-catenin target genes which are the drivers of the hallmarks of cancer [7-12]. Thus, nuclear expression of β-catenin is an indicator of active Wnt/β-catenin signalling and expectedly nuclear β-catenin is associated with tumour progression and poor prognosis in CC [13,14].

The transcription factor SOX2 is involved together with c-Myc, KLF4 and Oct3/4 in the induction and maintenance of pluripotent stem cells [15-18]. Recently it was demonstrated, that SOX2 is also expressed in human CCs. Similar to the expression of nuclear β-catenin, high SOX2 expression was associated with a poor prognosis, recurrence, and lower disease free survival of patients with CC [19,20]. In contrast, SOX2 was also shown to repress the transcriptional activity of the β-catenin/TCF complex in vitro using TOP-flash assays and thus, repressing the expression of TCF target genes [18,21,22]. Thus, SOX2 directly induces malignant progression of CCs and indirectly represses it at the same time via suppression of β-catenin activity. Currently it is unknown if SOX2 and β-catenin are coexpressed in CCs and if a coexpression has an influence on the progression. Finally, we wanted to clarify, if the poorer outcome of CCs with high SOX2 expression is associated with an increased occurrence of distant metastases, which is known to be the best predictor for an unfavourable course of tumour diseases [19,20]. Therefore, we employed a case control collection of human CCs with or without distant metastases applying immunohistochemistry.

## Methods

### Tissue collection

Formalin fixed-paraffin embedded (FFPE) samples of right-sided CCs from 114 patients that underwent curative surgical tumour resection at the Ludwig-Maximilians-Universität (LMU) München between 1994 and 2005 were recovered from the archives of the Institute of Pathology. The corresponding clinico-pathological datasets were obtained from the Munich Cancer Registry (MCR, Tumorzentrum München) (Table 1). For statistical reasons the collection was highly selected in the form of a two-armed case-control study. One arm of the collection consisted of CCs with synchronous liver metastases, where metastasis was diagnosed by clinical imaging or liver biopsy. The other arm of the collection was represented by CCs without distant metastases at the time of diagnosis and with a disease free survival of at least 5 years after primary surgical resection to exclude the development of metastases. Patients in both arms were matched with respect to tumour grade (according to WHO 2000), T stage (according to UICC 2009), and tumour localization (right side), resulting in 57 matched pairs. The study was performed according to the recommendations of the local ethics committee of the Medical Faculty of the LMU München.

**Table 1:** Clinico-pathological variables and correlations with SOX2 and β-catenin protein expression in the matched case-control collection of 114 CC patients. OR = odds ratio, CI = 95% confidence interval, Percent-values are given in parentheses.

Characteristic	Total (%)	SOX-2		OR (CI)	*p*	β-catenin (cytoplasmic)		OR (CI)	*p*	β-catenin (nuclear)		OR (CI)	*p*
										
		high	low			high	low			high	low		
All patients	114 (100)	24 (21.1)	90 (78.9)			104 (91.2)	10 (8.8)			38 (33.3)	76 (66.7)		

Age (median. 66.5) years													

≤ 66	57 (50)	11 (9.6)	46 (40.4)	1.2	0.646	56 (49.1)	1 (0.9)	0.1	**0.008**	22 (19.3)	35 (30.7)	0.6	0.23

≥67	57 (50)	13 (11.4)	44 (38.6)	(0.5-3.0)		48 (42.1)	9 (7.9)	(0.01-0.8)		16 (14.0)	41 (36.0)	(0.3-1.4)	

Gender													

Male	58 (50.9)	15 (13.2)	43 (37.7)	1.8	0.20	54 (47.4)	4 (3.5)	1.6	0.471	22 (19.3)	36 (31.6)	1.5	0.29

Female	56 (49.1)	9 (7.9)	47 (41.2)	(0.7-4.6)		50 (43.9)	6 (5.3)	(0.4-6.1)		16 (14.0)	40 (35.1)	(0.7-3.4)	

Tumor size (UICC)													
T1	2 (1.8)	0 (0)	2 (1.8)	-	0.280	2 (1.8)	0 (0)	-	0.662	1 (0.9)	1 (0.9)	-	0.607

T2	10 (8.8)	0 (0)	10 (8.8)			10 (8.8)	0 (0)			2 (1.8)	8 (7.0)		

T3	86 (75.4)	21 (18.4)	65 (57.0)			77 (67.5)	9 (7.9)			31 (27.2)	55 (48.2)		

T4	16 (14)	3 (2.6)	13 (11.4)			15 (13.2)	1 (0.9)			4 (3.5)	12 (10.5)		

Nodal status													

N0	52 (45.6)	5 (4.4)	47 (41.2)	4.2	**0.006**	49 (43.0)	3 (2.6)	0.5	0.299	15 (13.2)	37 (32.5)	1.5	0.35

N+	62 (54.4)	19 (16.7)	43 (37.7)	(1.4 - 12.1)		55 (48.2)	7 (6.1)	(0.1-2.0)		23 (20.2)	39 (34.2)	(0.7-3.2)	

Metastasis (liver)													

M0	57 (50)	7 (6.1)	50 (43.9)	3.0	**0.022**	52 (45.6)	5 (4.4)	1.0	1.0	11 (9.6)	46 (40.4)	3.8	**0.001**

M1	57 (50)	17 (14.9)	40 (35.1)	(1.1-3.0)		52 (45.6)	5 (4.4)	(0.3-3.7)		27 (23.7)	30 (26.3)	(1.6-8.7)	

Tumor grade (WHO)													

G2	44 (38.6)	7 (6.1)	37 (32.5)	-	**0.043**	43 (37.7)	1 (0.9)	-	0.265	16 (14.0)	28 (24.6)	-	0.567

G3	66 (57.9)	15 (13.2)	51 (44.7)			58 (50.9)	8 (7.0)			21 (18.4)	45 (39.5)		

G4	4 (3.5)	2 (1.8)	2 (1.8)			3 (2.6)	1 (0.9)			1 (0.9)	3 (2.6)		

### Immunohistochemistry

All immunohistochemical stainings were done using 5 μm serial tissue sections of FFPE tumour samples. For SOX2 specific immunohistochemistry first a heat-induced epitope retrieval was done applying Epitope Retrieval Solution (Novocastra, Leica Biosystems, United Kingdom) before adding primary SOX2 specific rabbit monoclonal antibody (1:50, SOX2 clone D6D9, Cell Signalling Technology, Danvers, MA) for 60 min at room temperature. Thereafter, a development step was introduced by adding detection-system (AEC Solution, Invitrogen, Carlsbad, CA) and subsequently substrate-chromogen containing AEC + system (DAKO, Germany) according to the respective manufacturer's protocols. Finally, slides were counterstained using Hematoxylin (Vector Laboratories, Burlingame, CA). To control for unspecific binding of antibodies, isotype controls were included. Normal stomach tissues with a well described SOX2 staining pattern were used as the positive controls [23].

For the detection of β-catenin prediluted anti-β-catenin mouse monoclonal antibody (ready to use, clone 14; Ventana Medical Systems, Oro Valley, AZ) was used as the primary antibody. Staining was done employing a Ventana Benchmark XT autostainer using the XT UltraView diaminobenzidine kit (Ventana Medical Systems) following the manufacturer's instructions.

### Scoring of Immunohistochemistry

Evaluation of SOX2 immunostaining was done by two observers (JN, FB) separately for the nucleus and the cytoplasm. The results for nuclear staining were evaluated for both, percentage and intensity of stained nuclei. Intensity values were categorized as follows: no staining - 0, weak - 1, moderate - 2, and strong - 3. When less than 10% of cells were stained and/or the intensity was missing (0) or weak (1), immunostaining was reported to be negative [24]. The cytoplasm of cancerous colon tissue showed none or only weak staining for SOX2. Therefore, since SOX2 is known to be a nuclear transcription factor, cytoplasmic staining was not considered for further statistical analysis.

Results of the β-catenin specific immunohistochemistry were also evaluated by two observers (JN, LK) separately for the subcellular localization (cytoplasm and nucleus). For nuclear β-catenin expression the quantity of stained tumour cells throughout the whole tumour but not the intensity of staining was considered. The score for nuclear β-catenin expression was as follows: negative - 0, < 30% - 1+, 30-60% - 2+, > 60% positive cells - 3+. For cytoplasmic β-catenin expression the intensity of specific staining was analysed and graded into no - 0, weak - 1, moderate - 2 or strong cytoplasmic staining - 3. For subsequent statistical analysis the cases were classified into low (scores 0 and 1) and high grade (scores 2 and 3) extent for nuclear and cytoplasmic protein expression, respectively.

### Statistics

Significance of correlations of the immunhistochemical analyses were calculated using the χ^2^-test using SPSS v. 18.0 (SPSS Inc., Chicago, IL). For all tests a two-sided α-error of less than 5% (p < 0.05) was considered statistically significant.

## Results

**Figure 1:**
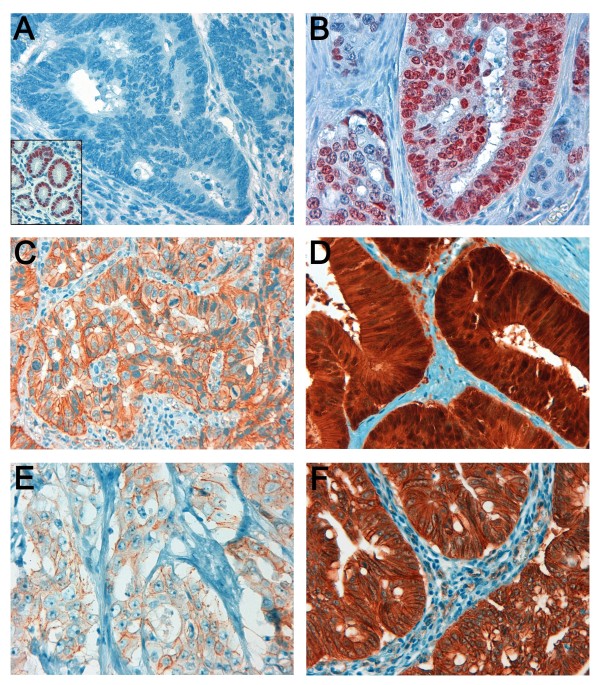
**Immunohistochemical staining of SOX2 (**A **and **B**) and β-catenin (**C - F**) in colonic adenocarcinoma (400-fold magnification)**. CC with absence of SOX2 expression (**A**). Normal gastric mucosa was used as the positive control (insert). Tumours with presence of moderate or strong nuclear protein expression of SOX2 in more than 10% of tumour cells were classified as SOX2 positive (**B**). Tumour cells with absence (**C**) or presence (**D**) of nuclear β-catenin accumulation were classified as β-catenin low or β-catenin high, respectively. Weak (1+) and strong (3+) cytoplasmic staining for β-catenin are shown in (**E**) and (**F)**, respectively.

To investigate the expression patterns of SOX2 and β-catenin in CC and to correlate these with the occurrence of metastases serial sections of 114 patients either with metastases (N = 57) or without metastases and a disease free survival of more than 5 years (N = 57) was subjected to immunohistochemistry (Figure 1). Based on the immunohistochemical score published by Park et al., a total of 21.1% of tumours showed nuclear SOX2 expression [24]. Expression of SOX2 correlated significantly with lymph node metastases (*p *= 0.006), distant metastases (*p *= 0.022) and histopathological tumour grade (*p *= 0.043) according to WHO. No correlations with tumour size (according to UICC), gender, or age were found (Table 1). Absence of SOX2 expression was associated with an advanced T-category (T3 - T4) but reduced rates of locoregional- and distant spread and lower histopathological tumour grades (G2 - G3; Table 1). In cases with low SOX2 but high β-catenin expression a higher rate of distant metastases could be obtained (Table 2).

**Table 2:** Correlation of combined protein expression scores of SOX2 and nuclear β-catenin expression with lymph-node metastases and distant spread. Percent-values are given in parentheses.

	Nodal status			Distant metastases		
				
Protein expression profile	N0	N+	*p*	M0	M1	*p*
SOX2 and β-catenin low	32 (28.1)	28 (24.6)	**0.02**	40 (35.1)	20 (17.5)	**0.001**

SOX2 low and β-catenin high	15 (13.2)	15 (13.2)		10 (8.8)	20 (17.5)	

SOX2 high and β-catenin low	5 (4.4)	11 (9.6)		6 (5.3)	10 (8.8)	

SOX2 and β-catenin high	0 (0)	8 (7.0)		1 (0.9)	7 (6.1)	

**Figure 2:**
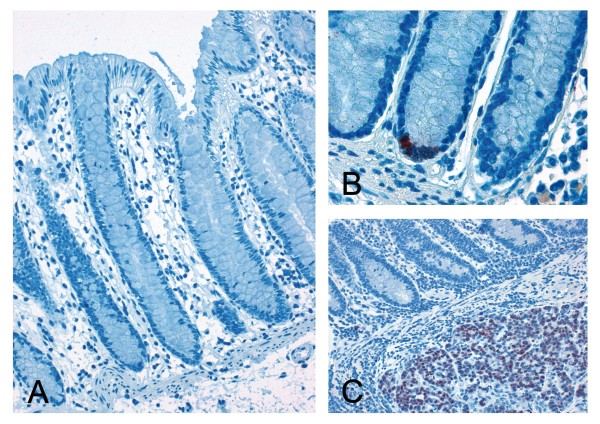
**Immunohistochemical staining of SOX2 in normal colonic mucosa.** Normal colonic mucosa with absent SOX2 expression (**A**, 200-fold magnification). Few cases (4%) reveal a positive SOX2 expression within the stem-cell niche of the colonic crypt (**B**, 630-fold). Also adjacent to positive tumour cell infiltrates no SOX2 expression could be detected in the normal colonic mucosa in the majority of cases (**C**, 200-fold).

In 83 cases adjacent normal colonic tissue was available. Of these only four cases (4.8%) showed nuclear expression within the stem cell niche of the colonic crypts (Figure 2). No correlation of the expression patterns of SOX2 in the normal and cancerous tissue could be obtained, respectively.

Strong nuclear β-catenin expression was found in 33.3% of cases (N = 38) and correlated significantly with the occurrence of distant metastases (*p *= 0.001), but not with lymph node metastases, tumour stage, histopathological tumour grade, gender, or patient's age (Table 1). High levels of cytoplasmic protein expression of β-catenin (N = 104; 91.2%) correlated only with the age of patients. No correlation with patients' gender, tumour size, nodal status, distant spread or tumour grade emerged (Table 1).

Combined high scores of SOX2 and nuclear β-catenin expression were present in 8 of 114 cases (7.0%), thus representing about one third of all SOX2 positive cases. In this subgroup a significant correlation with nodal status (100% showed lymph-node metastases, *p *= 0.02) and distant spread (seven of eight cases (87.5%) showed distant metastases, *p *= 0.001; Table 2). Absence of SOX2 and nuclear β-catenin expression was associated with a reduced rate of distant metastases (only 33.3% of these patients showed distant spread). No impact on the frequency of lymph-node metastases could be derived in this group of CC patients (Table 3).

**Table 3:** Correlation of SOX2 protein expression and nuclear and cytoplasmic β-catenin protein expression in the matched case-control collection of 114 CC patients. Percent-values are given in parentheses.

	SOX-2		
		
	high	low	*p*
**β-catenin (cytoplasmic)**			

**Score 0**	0 (0)	0 (0)	0.462
	
**Score 1**	2 (1.8)	8 (7.0)	
	
**Score 2**	8 (7.0)	42 (36.8)	
	
**Score 3**	14 (12.3)	40 (35.1)	

**low (0 + 1)**	2 (1.8)	8 (7.0)	0.932
	
**high (2 + 3)**	22 (19.3)	82 (71.9)	

**β-catenin (nuclear)**			

**Score 0**	6 (5.3)	29 (25.4)	0.897
	
**Score 1**	10 (8.8)	31 (27.2)	
	
**Score 2**	5 (4.4)	19 (16.7)	
	
**Score 3**	3 (2.6)	11 (9.6)	

**low (0 + 1)**	16 (14.0)	60 (52.6)	1.0
	
**high (2 + 3)**	8 (7.0)	30 (26.3)	

Nevertheless, no correlation between SOX2 expression and nuclear or cytoplasmic β-catenin protein levels with each other was observed (Table 3, SOX2 versus nuclear β-catenin: *p *= 1.0; OR = 1.0, 95% CI = 0.4-2.6 and SOX2 versus cytoplasmic β-catenin: *p *= 0.932; OR = 1.1, 95% CI = 0.2-5.4).

## Discussion

In our two armed, matched collection of CCs we demonstrated that high levels of both SOX2 and nuclear β-catenin expression, respectively, were associated significantly with metastatic disease. This observation might explain the association of SOX2 protein expression with poorer prognosis and reduced overall survival in CC, reported in the literature [19,20]. Lymph node and distant metastases are both independent prognostic factors correlating with poor survival in CC [25]. Patients with node-negative CCs (UICC stage I/II) have a 5 year survival exceeding 75%, whereas patients with node-positive disease only have a 5 year survival rate of less than 50% [26]. Similar, patients with synchronous- or metachronous liver metastases reveal 5 year survival rates following liver resection ranging between 25% and 55% whereas for non-operated patients this rate lies between 0% and 5% [27].

The highest rate of lymph node- and distant metastases could be obtained in the group of CC with combined scores of high SOX2 and high β-catenin expression, whereas tumours with low or absent expression of both markers were associated with a reduced risk for loco-regional- and distant spread. SOX2 negative cases with high nuclear β-catenin expression showed higher rates of distant metastases but no association with the nodal status. Since nuclear β-catenin is involved in epithelial-mesenchymal transition (EMT) and stem cell formation during embryonic development, the combination of EMT and stem cell competence during malignant progression of CC, both regulated by β-catenin, may result in "migrating tumour stem cells", driving tumour invasion and metastases [6,9]. β-catenin is considered to be one of the driving forces for the initiation and maintenance of colorectal cancer stem cells (CSCs). In addition, SOX2 is an inducer of embryonic stemness which is part of the signature of colorectal CSCs [28,29]. Mechanistically, it might therefore be assumed that both β-catenin and SOX2 drive the malignant progression of CCs by inducing cancer stemness of colorectal tumour cells. CSCs are known to be responsible for the process of metastasis [30].

This is also supported by our finding that SOX2 expression can be found within the stem cell niche in a small proportion of normal colonic crypts (Figure 2). The fact, that SOX2 plays a major role for the development of the upper but not of the lower digestive tract may explain why SOX2 protein-expression can only be found in a minority of cases within the normal colonic tissue. This finding is concordant to previously published data [19,21]. To further investigate this issue we correlated the SOX2 expression with previously published data on CD133, an established marker for CSC which is predictive for poor prognosis and distant spread in CC [31-33]. Unexpectedly, no significant correlation between SOX2 and CD133 expression could be demonstrated in our collection on CC (χ^2^-test, data not shown). However, similar to β-catenin combined scores of high SOX2 and high CD133 showed strong predictive value according to the risk of distant spread (8 of 10 cases; 80%) and the presence of lymph-node metastases (9 of 10 cases; 90%). In summary, our data underline the importance of stemness-associated markers for the prediction of distant spread and the prognostification of CC. These observations also indicate that markers and marker-combinations for a stemness phenotype could be relevant for identification of CCs with high risk for distant spread and subsequently could have major impact on clinical decisions according to the radicalness of loco-regional surgery and adjuvant chemotherapy.

Through in vitro studies it was shown that SOX2 represses the transcriptional activity of the β-catenin/TCF complex, thus affecting the expression of oncogenic β-catenin driven transcription [18,21,22]. The assumed underlying mechanism, as far as already understood, is complex as SOX2 directly binds to the β-catenin/TCF complex and vice versa SOX2 transcription is regulated by β-catenin [34-36]. Moreover, knockdown of SOX2 decreased the growth rate of cultivated colorectal tumour cell lines in vitro and in vivo [37]. Surprisingly, no correlation of SOX2 and β-catenin protein expression levels could be demonstrated in our collection of CRCs. Nevertheless, it could not be concluded from our results that the reported negative regulatory feedback between β-catenin and SOX2 does not occur in human CRC [34,38-40]. Therefore, to clarify this issue, further immunohistochemical and functional studies involving SOX2 and Wnt-regulated target genes are needed to elaborate the actual impact of the regulatory feedback mechanisms between SOX2 and the β-catenin/TCF-complex in CRC patients.

Taken together, our results suggest that SOX2 protein expression may be a possible prognostic marker in the management of CC patients. Nevertheless, this observation requires validation in larger, randomized patient cohorts including also the left side of the colon and the rectum before SOX2 immunohistochemistry should be used in the clinical setting.

## Conclusion

In summary, we report that overexpression of SOX2 significantly correlated with lymph-node and distant metastases in right-sided CCs. Since both types of metastases are independent predictors of bad prognosis our results are in concordance with the reduced survival reported in the literature. In the same collection, we revealed that nuclear β-catenin was associated with distant metastases. Furthermore, coexpression of both markers was significantly associated with distant spread and lymph-node metastases in our collection of CCs.

## Competing interests

The authors declare that they have no competing interests.

## Authors' contributions

JN: Participated in the design and coordination of the study, evaluated the β-catenin and SOX2 immunohistochemistry, performed the statistical analysis and drafted the manuscript. FB: Evaluated the SOX2 immunohistochemistry, participated in the selection of the case control collection. DH: Participated in the design and selection of the case control collection and helped to draft the manuscript. LK: Participated in the selection of the case control collection and evaluated the β-catenin immunohistochemistry. JE: Participated in the design of the case control collection and provided clinical data from the Munich Cancer Registry. RML: Validated the SOX2 antibody for immunohistichemistry. MG: Participated in the design and coordination of the study and helped to draft the manuscript. TK: Conceived of the study, participated in the design, provided funding and helped to draft the manuscript. AJ: Is the principal investigator, conceived of the study and participated in its design and coordination, provided funding and drafted the final manuscript. All authors read and approved the final manuscript.

## Pre-publication history

The pre-publication history for this paper can be accessed here:

http://www.biomedcentral.com/1471-2407/11/518/prepub
